# Large-scale Gene Ontology analysis of plant transcriptome-derived sequences retrieved by AFLP technology

**DOI:** 10.1186/1471-2164-9-347

**Published:** 2008-07-24

**Authors:** Alessandro Botton, Giulio Galla, Ana Conesa, Christian Bachem, Angelo Ramina, Gianni Barcaccia

**Affiliations:** 1 Department of Environmental Agronomy and Crop Science, University of Padova, Viale dell'Università 16, Campus of Agripolis, 35020 Legnaro, Italy; 2 Bioinformatics Department, Centro de Investigaçión Príncipe Felipe, Avda. Autopista Saler 16, 46013 Valencia, Spain; 3 Department of Plant Sciences, Laboratory of Plant Breeding, Wageningen University and Research Centrum, PO Box 386, 6700 AJ Wageningen, The Netherlands

## Abstract

**Background:**

After 10-year-use of AFLP (Amplified Fragment Length Polymorphism) technology for DNA fingerprinting and mRNA profiling, large repertories of genome- and transcriptome-derived sequences are available in public databases for model, crop and tree species. AFLP marker systems have been and are being extensively exploited for genome scanning and gene mapping, as well as cDNA-AFLP for transcriptome profiling and differentially expressed gene cloning. The evaluation, annotation and classification of genomic markers and expressed transcripts would be of great utility for both functional genomics and systems biology research in plants. This may be achieved by means of the Gene Ontology (GO), consisting in three structured vocabularies (*i*.*e*. ontologies) describing genes, transcripts and proteins of any organism in terms of their associated cellular component, biological process and molecular function in a species-independent manner. In this paper, the functional annotation of about 8,000 AFLP-derived ESTs retrieved in the NCBI databases was carried out by using GO terminology.

**Results:**

Descriptive statistics on the type, size and nature of gene sequences obtained by means of AFLP technology were calculated. The gene products associated with mRNA transcripts were then classified according to the three main GO vocabularies. A comparison of the functional content of cDNA-AFLP records was also performed by splitting the sequence dataset into monocots and dicots and by comparing them to all annotated ESTs of Arabidopsis and rice, respectively. On the whole, the statistical parameters adopted for the *in silico *AFLP-derived transcriptome-anchored sequence analysis proved to be critical for obtaining reliable GO results. Such an exhaustive annotation may offer a suitable platform for functional genomics, particularly useful in non-model species.

**Conclusion:**

Reliable GO annotations of AFLP-derived sequences can be gathered through the optimization of the experimental steps and the statistical parameters adopted. The Blast2GO software was shown to represent a comprehensive bioinformatics solution for an annotation-based functional analysis. According to the whole set of GO annotations, the AFLP technology generates thorough information for angiosperm gene products and shares common features across angiosperm species and families. The utility of this technology for structural and functional genomics in plants can be implemented by serial annotation analyses of genome-anchored fragments and organ/tissue-specific repertories of transcriptome-derived fragments.

## Background

The advances in high-throughput molecular biology technologies of the last two decades have not only led to a drastic change in our ability to study genomes, but also brought together novel mechanisms for structuring, storing and sharing information. Concerning gene annotation, previous scenarios of nomenclature diversity and large efforts for gathering all available information on a given species have been replaced by the availability of standard vocabularies extensively used by the scientific community. This, along with the availability of user-friendly bioinformatic tools, allows the feasible evaluation, functional annotation and classification of a high number of expressed sequences in a great variety of organisms. Such characterization would be useful for functional genomics research in plants, particularly in the emergent field of systems biology. With the progress of plant genome sequencing projects, in-depth knowledge about molecules, such as nucleic acids and deduced proteins, gene regulatory networks, and metabolic pathways becomes possible. Hierarchically structured ontological terms can now be adopted to query sequences and to describe genes and their products at different levels of knowledge and specificity [1].

The Gene Ontology (GO) project began in 1998 with the integration of three model organism databases, *i*.*e*. yeast, *Drosophila *and mouse, and represents today the most widely used schema for the functional characterization of plant, animal and microbial genes and gene products [1]. The GO project has developed three structured vocabularies (*i*.*e*. ontologies) describing genes, transcripts and proteins of any organism in terms of their associated cellular component, biological process and molecular function in a species-independent manner. The use of GO terms by collaborating databases facilitates uniform retrievals across them. Moreover, the GO vocabularies can be queried at different levels, allowing annotators to assign properties to genes or gene products, depending on the depth of knowledge and specificity about that entity [2].

In plant species for which the genome sequence is not available, AFLP and cDNA-AFLP are two of the most commonly used methods for genome- and transcriptome-wide level analysis (see Additional file 1), respectively, capable of discovering genes not yet been cloned or even predicted, based upon their polymorphisms or differential expression patterns.

AFLP markers [3-5] represent the genomic tool with the highest polymorphism information content, and combine the reliability of the RFLP technique with the power of the PCR technique. Both AFLP and AFLP-derived markers, such as M-AFLPs (Microsatellite-AFLPs) and S-SAPs (Sequence-Specific Amplified Polymorphisms), have been widely used for whole genome scanning and fingerprinting [6-13], characterizing single chromosomes and mapping specific genes [14-20], as well as for transcriptome profiling and gene cloning by means of cDNA-AFLP [21-23], and to generate quantitative gene expression patterns for eQTL mapping, as recently demonstrated by Vuylsteke et al. [24].

Transcriptome differential profiling of plants with antagonistic phenotypes (*e*.*g*. mutant vs. wildtype) is theoretically one of the most powerful strategies for identifying and cloning candidate genes not only for model organisms, but also in remarkably complex genomes such as polyploids [25-32].

The mRNA fingerprinting based on AFLP technology does not require pre-existing genome or EST sequence knowledge, therefore it is being widely used in less well investigated systems. Since a few genomes of agriculturally important species (*e.g*., rice, poplar and grapevine) have been sequenced and even fewer have been well annotated, the data provided by cDNA-AFLP experiments may represent a valuable resource for functional genomics and genetics in non-model plants. Compared to microarrays, cDNA-AFLP increases the resolution of expression patterns detection using smaller amounts of mRNA [33]. This feature is essential when an RNA fingerprinting is applied to tissues for which it is hard to isolate stage-specific messengers, such as flowers, fruits and seeds. Both reliability and sensitivity of amplification products proved to be very high, and expression patterns visualized by cDNA-AFLP showed to well correlate with northern blot analyses [26,27,34-37]. Moreover, the redundancy of the technique [38] proved to be very informative in cases of alternative splicing and multigene family member displaying [27,29,32], for distinguishing highly homologous genes [39-41].

After 10-year-use of AFLPs for DNA fingerprinting and mRNA profiling, large sequence collections retrieved with this technology are available in public databases for several crop and model species. The evaluation, annotation and classification of AFLP-derived genomic markers and expressed transcripts would be very useful for both functional genomics and systems biology research in plants, and crucial to allow the scientific community to promptly retrieve this pre-existing information from gene banks. Annotations could be periodically revised and implemented, and should allow to optimize genomic AFLP and cDNA-AFLP experiments in plant species to give specific information about the targeted biological processes and molecular functions.

In the present study, we retrieved a total of 7,806 cDNA-AFLP sequences related to roots, leaves, stems, flowers, fruits and seeds from both NCBI databases and unpublished repertories. All these entries belong to 22 different species distributed among seven botanic families: *Solanaceae*, *Fabaceae*, *Poaceae*, *Salicaceae*, *Rosaceae*, *Brassicaceae *and *Vitaceae*. Redundant sequences were clustered and contigs assembled. Functional analysis was then performed using Blast2GO [42]. Blast2GO is a bioinformatic tool for the GO-based annotation and data mining of sequence sets for which no GO annotation is yet available, which has proven to be effective in the functional characterization of plant sequence data [43]. The cDNA-AFLP-derived sequences were then grouped according to the GO vocabularies. Experimental steps and statistical parameters adopted for the *in silico *analysis were critical for obtaining reliable gene ontology data. Annotation results for the whole sequence dataset and also for botanic families, single species and plant organs are presented, and the main features of genes and gene products detectable in plants by AFLP technology discussed.

## Results

### Gene Ontology of AFLP-derived cDNA sequences

The total number of plant cDNA sequences derived from AFLP technology deposited in NCBI databases or recovered from private unpublished collections was as high as 7,806. Entries belonged to 22 different model, crop and tree species distributed among seven botanic families: *Solanaceae *(3,734), *Salicaceae *(1,003), *Fabaceae *(975), *Poaceae *(906), *Rosaceae *(769), *Brassicaceae *(262) and *Vitaceae *(226). Additional 69 cDNA-AFLP-derived sequences of *Salix *spp. were analyzed separately once they became publicly available because although deposited they were not released at the time of entries downloading. The most abundant taxonomic group included in the present study was the genus *Nicotiana*, followed by *Populus, Medicago*, *Oryza *and *Malus*. The list of organisms with their corresponding number of cDNA-AFLP sequences deposited in NCBI databases is reported in the Methods section (see Table 1).

**Table 1:** cDNA-AFLPs redundancy.

Species	Sequences		Contigs	Singlets		Redundancy	GC content
					
	No.	%	No.	No.	%	%	%
*Aegilops tauschii*	115	1.6	19	73	63.5	36.5	46.4
*Arabidopsis *spp.	155	2.2	4	147	94.8	5.2	43.3
*Brassica napus*	107	1.5	3	101	94.4	5.6	46.2
*Cicer arietinum*	48	0.7	5	37	77.1	22.9	43.4
*Fragaria ananassa*	61	0.9	0	61	100.0	0.0	46.7
*Hordeum vulgare*	85	1.2	6	73	85.9	14.1	50.2
*Lolium perenne*	34	0.5	0	34	100.0	0.0	51.7
*Lotus japonicum*	148	2.1	3	142	95.9	4.1	41.3
*Lysopersicon esculentum*	227	3.2	24	161	70.9	29.1	41.8
*Malus domestica*^1^	525	7.4	47	409	77.9	22.1	45.7
*Medicago *spp.	681	9.6	25	625	91.8	8.2	41.4
*Nicotiana *spp.	2,995	42.3	308	2,235	74.6	25.4	42.3
*Oryza sativa*	598	8.4	58	461	77.1	22.9	45.6
*Phaseolus vulgaris*	98	3.0	7	82	83.7	16.3	44.9
*Petunia hybrida*	212	1.4	14	181	85.4	14.6	42.2
*Populus *spp.	865	12.2	97	624	72.1	27.9	44.4
*Prunus persica*	94	1.3	11	57	60.6	39.4	45.2
*Prunus avium*	89	1.3	16	51	57.3	42.7	43.9
*Salix *spp.^2^	69	1.0	8	61	88.4	11.6	44.2
*Solanum *spp.	300	4.2	52	167	55.7	44.3	42.6
*Tirticum aestivum*	74	1.0	7	56	75.7	24.3	48.3
*Vitis vinifera*^3^	226	3.2	16	180	79.6	20.4	47.1

Overall	7,806		730	6,018	77.1	22.9	44.9

Redundancy information on cDNA-AFLP sequences assessed on the basis of the number and proportion of contigs and singlets. For each sequence subset, the number of retrieved sequences and the percentage of the total, the number of contigs, the number of singlets and the percentage of the total, the redundancy, and the GC content are reported.

^1^278 sequences deposited in GenBank but not yet publicly released; ^2^All 69 sequences were released after February 1^th^, 2007; ^3^113 sequences are not yet publicly available.

Redundant cDNA-AFLP sequences for each of the 22 organisms were clustered into a total of 730 contigs, whereas the remaining 6,018 records (77.1% of the total raw sequences) were singlets. The overall estimate of redundancy was about 23%, ranging from 0% in *Fragaria *and *Lolium*, to 44.3% of *Solanum *spp, whereas the GC content of cDNA-AFLP sequences was found to be 45%, with a standard deviation over all organisms as low as 2.8%. Redundancy statistics and GC contents for all organisms are summarized in Table 1.

With the exception of the 69 *Salix *entries (analyzed separately from the rest of entries by Quaggiotti et al. [32] because they were released after February 1^st^, 2007), the 6,679 cDNA-AFLP sequences equal to 722 contigs plus 5,957 singlets and belonging to 21 *Angiospermae *were used as BlastX queries to search for structural homologies and significant similarities. A total of 4,332 sequences (64.9%) revealed significant similarity with deposited records, showing an average nucleotide similarity estimate of 80% and a median E-value of 1 e-13 over all organisms (see Additional file 2). The same statistics were also computed for each single species or genus (see Additional file 3).

**Figure 1:**
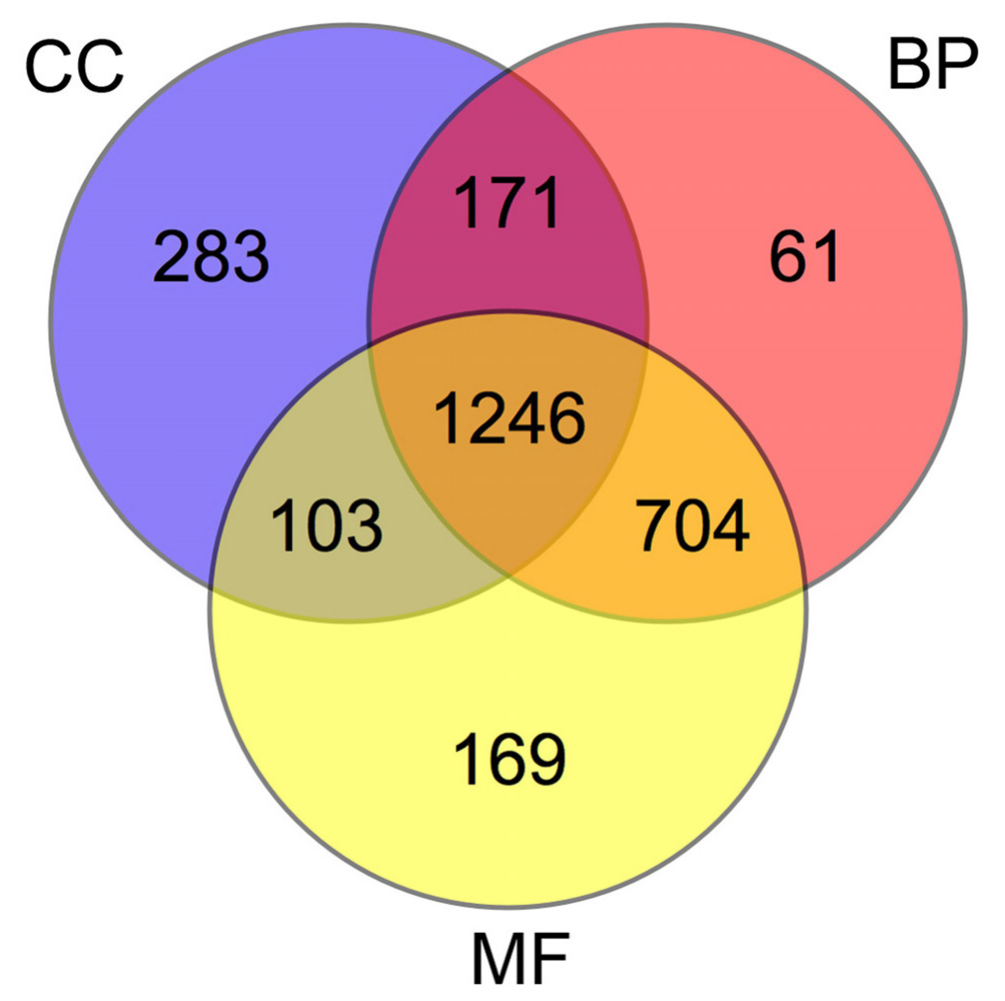
**cDNA-AFLPs Venn diagram**. Venn diagram of cDNA-AFLP sequences annotated by one, a combination of two and all three GO vocabularies.

GO term mapping allowed the identification of 11,409 GO terms based upon 4,332 matches retrieved by BlastX. The distribution of annotations among GO vocabularies and the number of cDNA-AFLP sequences with GO terms belonging to only one, a combination of two and all three vocabularies was organized in a Venn diagram (Fig. 1). 1,246 sequences were annotated according to all the three GO subvocabularies (*i.e*. 'cellular component', CC; 'biological process', BP; 'molecular function', MF) and annotations simplified using a plant-specific GOslim following the approach suggested by similar papers with comparative purposes [44-46].

As many as 704, 171 and 103 sequences were annotated with a combination of BP and MF terms, BP and CC terms, and CC and MF terms, respectively.

Basic statistics for all the cDNA-AFLP annotated sequences sorted by botanic family and by single organism, including mean length and length range for a 68% confidence interval, mean similarity and similarity range for a 68% confidence interval, median E-value with minimum and maximum E-values are reported in Tables 2 and 3.

**Table 2:** Annotated cDNA-AFLPs statistics by botanic family.

		**Length (bp)**			**Similarity (%)**			**E-value**	
		
Family	Total No.	No.^†^	Mean^‡ ^(conf. int.)^†^	CV (%)^‡^	No.^†^	Mean^‡ ^(conf. int.)^†^	CV (%)^†^	Median^‡^	min^‡ ^– max^‡^
*Brassicaceae*	92	62	237 (118 – 356)	50	78	87 (75 – 100)	14	6e-18	1e-95 – 1e+00
*Fabaceae*	449	354	238 (102 – 375)	57	301	81 (69 – 93)	15	1e-15	1e-122 – 1e+00
*Poaceae*	410	372	255 (56 – 454)	78	307	86 (74 – 98)	14	1e-20	1e-170 – 1e+00
*Rosaceae*	373	255	318 (192 – 444)	40	247	83 (71 – 94)	14	1e-29	1e-135 – 1e+00
*Salicaceae*	357	250	206 (112 – 300)	46	244	85 (74 – 96)	13	1e-15	1e-93 – 1e+00
*Solanaceae*	960	829	243 (76 – 410)	69	642	83 (71 – 95)	15	1e-15	1e-170 – 1e+00
*Vitaceae*	126	90	301 (170 – 431)	43	79	80 (67 – 93)	16	6e-23	1e-104 – 1e+00

All Organisms	2,767	2,212	252 (97 – 407)	62	1,898	83 (71 – 95)	14	1e-18	1e-170 – 1e+00

Statistics on cDNA-AFLP annotated sequences sorted by botanic family.

^†^Refers to the number of sequences included in the 68% confidence interval. ^‡^Refers to the total number of sequences. Abbreviations: conf. int., confidence interval; CV, coefficient of variability; min, minimum; max, maximum).

**Table 3:** Annotated cDNA-AFLPs statistics by organism.

**Organism**		**Length (bp)**			**Similarity (%)**			**E-value**	
		
	Total No.	No.^†^	Mean^‡ ^(conf. int.)^†^	CV (%)^‡^	No.^†^	Mean^‡ ^(conf. int.)^†^	CV (%)^‡^	Median^‡^	min^‡ ^– max^‡^
*Aegilops tauschii*	38	33	225 (149 – 301)	34	27	82 (69 – 94)	15	1e-14	1e-43 – 1e+00
*Arabidopsis *spp.	47	34	236 (139 – 334)	41	38	89 (78 – 99)	12	1e-22	1e-80 – 1e+00
*Brassica *spp.	41	27	219 (88 – 350)	60	31	90 (81 – 99)	10	1e-16	1e-93 – 1e+00
*Cicer arietinum*	18	16	219 (78 – 359)	64	14	90 (82 – 98)	9	5e-13	1e-66 – 1e+00
*Fragaria ananassa*	41	35	291 (139 – 442)	52	24	84 (74 – 93)	12	1e-43	1e-135 – 1e+00
*Hordeum vulgare*	46	42	425 (0 – 890)	110	34	83 (72 – 95)	14	1e-31	1e-170 – 1e+00
*Lolium multiflorum*	13	9	192 (113 – 271)	41	8	87 (78 – 96)	10	1e-09	1e-51 – 1e+00
*Lotus japonicus*	56	54	183 (69 – 296)	62	43	85 (74 – 96)	13	6e-11	1e-86 – 1e+00
*Lycopersicon esculentum*	93	72	173 (94 – 253)	46	63	84 (73 – 96)	13	1e-13	1e-78 – 1e+00
*Malus domestica*	246	165	341 (209 – 472)	39	167	82 (70 – 95)	15	6e-31	1e-109 – 1e+00
*Medicago *spp.	275	201	256 (118 – 393)	54	190	83 (71 – 95)	14	1e-17	1e-115 – 1e+00
*Nicotiana *spp.	722	661	240 (61 – 418)	74	489	83 (71 – 95)	15	1e-13	1e-170 – 1e+00
*Oryza sativa*	279	39	424 (351 – 498)	17	189	88 (78 – 98)	11	1e-20	1e-98 – 1e+00
*Petunia hybrida*	77	54	281 (161 – 400)	42	51	85 (74 – 95)	12	1e-22	1e-75 – 1e+00
*Phaseolus vulgaris*	40	25	185 (126 – 245)	32	27	85 (74 – 96)	13	5e-13	1e-40 – 1e+00
*Populus *spp.	333	231	206 (113 – 300)	45	229	85 (74 – 96)	13	1e-15	1e-93 – 1e+00
*Prunus avium*	41	28	230 (147 – 313)	36	29	84 (75 – 92)	11	1e-16	1e-47 – 1e+00
*Prunus persica*	54	40	332 (239 – 426)	28	34	79 (65 – 93)	18	1e-34	1e-78 – 1e+00
*Salix *spp.	24	18	204 (101 – 308)	50	14	84 (73 – 95)	13	1e-11	1e-62 – 1e+00
*Solanum *spp.	124	88	261 (122 – 400)	53	81	82 (71 – 93)	14	1e-16	1e-109 – 1e+00
*Triticum aestivum*	28	19	243 (145 – 341)	40	20	84 (72 – 96)	14	6e-20	1e-53 – 1e+00
*Vitis vinifera*	126	90	301 (170 – 431)	43	79	80 (67 – 93)	16	6e-23	1e-104 – 1e+00

Statistics on cDNA-AFLP annotated sequences sorted by organism.

^†^Refers to the number of sequences included in the 68% confidence interval. ^‡^Refers to the total number of sequences. Abbreviations: conf. int., confidence interval; CV, coefficient of variability; min, minimum; max, maximum.

**Figure 2:**
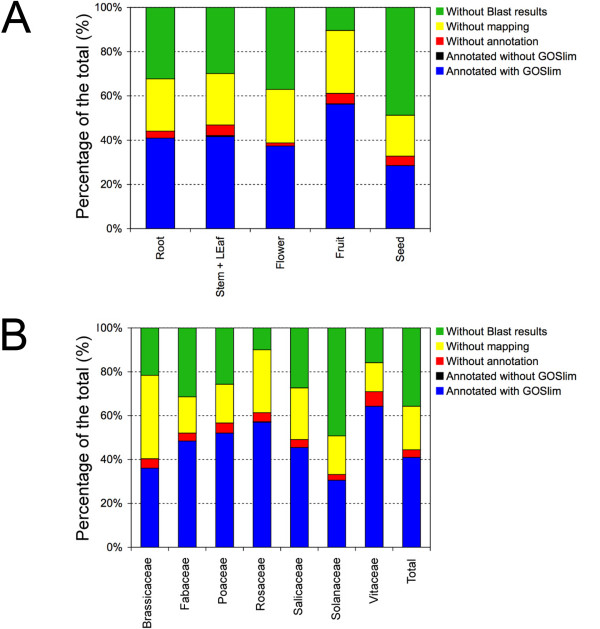
**Data distribution of cDNA-AFLP records**. Data distribution of sequences grouped according to the organ/tissue/part of the plant (A) and botanic family (B). Without Blast results: giving no significant similarity in BlastX analysis; without mapping: no GO term was mapped according to the information found in the blast matches; without annotation: the mapped GO terms were not reliable or without a significant score; annotated without GOslim: the GO annotation had no specific matching GO term in the GOslim used; annotated with GOslim: sequences with specific GOslim terms.

Among the botanic families, *Vitaceae *showed the highest proportion of annotated sequences (over 60%) and *Rosaceae *the lowest without Blast matches (less than 10%). On the whole, 36% of sequences did not retrieve any Blast result within the set E-value threshold. Mapping of GO terms and annotation were not possible for 20% and 3% of sequences, respectively, whereas the remaining 41% was annotated. Overall data distribution of cDNA-AFLP sequences grouped according to the organ, tissue or part of the plant, and botanic family showed a large variation for the percentage of sequences either with no Blast results or annotated, whereas the percentage of sequences neither with mapping nor annotation was quite constant (Fig. 2). Fruits were the organs with the highest percentage of annotated sequences and the lowest percentage without Blast matches, whereas the opposite was for seeds. Similar properties were observed for roots, stems and leaves, and flowers.

### Level 2 GOslim analysis of AFLP-derived cDNA sequences

**Figure 3:**
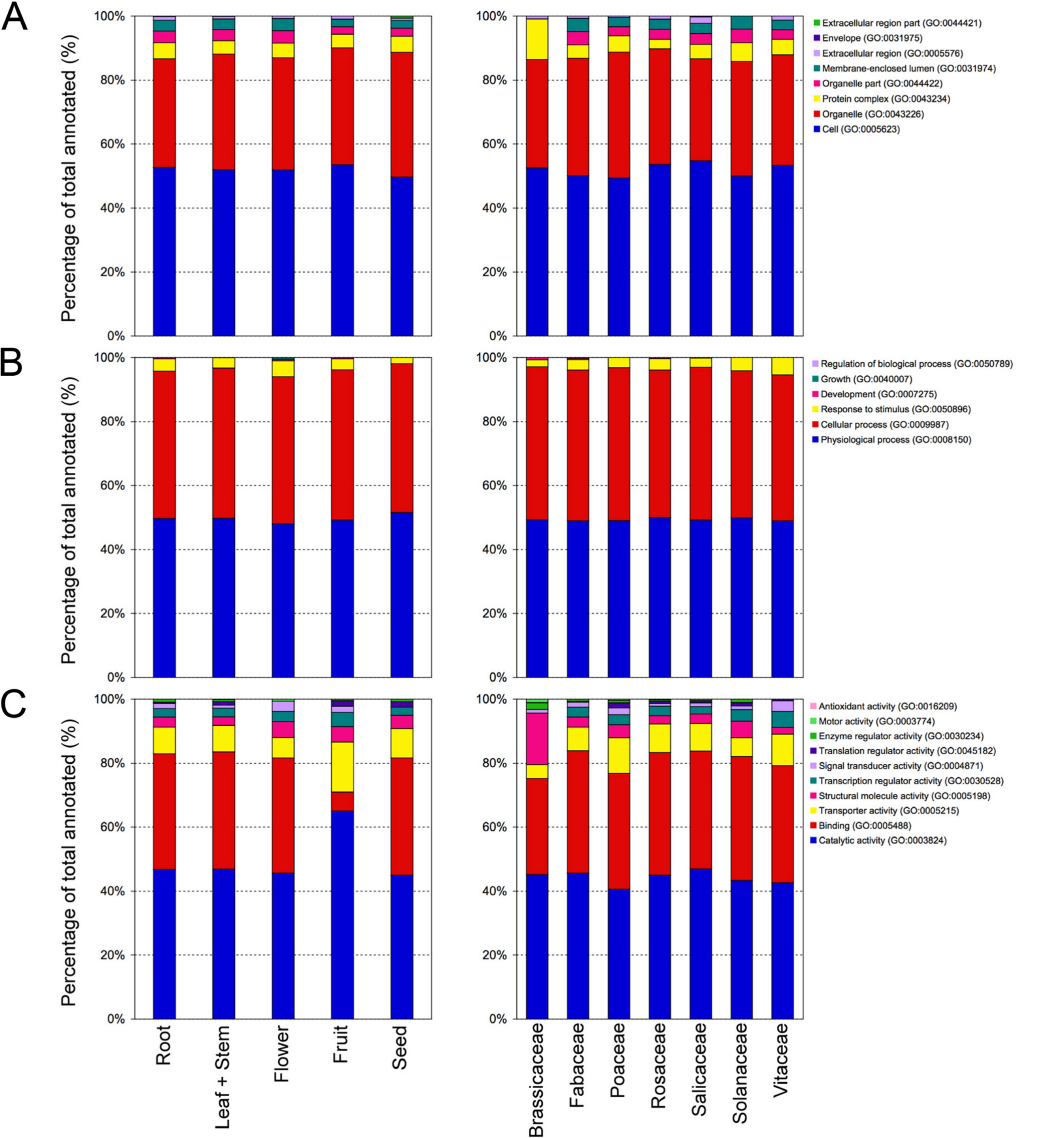
**Level 2 cDNA-AFLPs GO classification**. GOslim classification of annotated cDNA-AFLP sequences at level 2 for the three main GO vocabularies: cellular component (A), biological process (B) and molecular function (C). Sequences grouped according to the organ/tissue/part of the plant (left) and botanic family (right).

**Figure 4:**
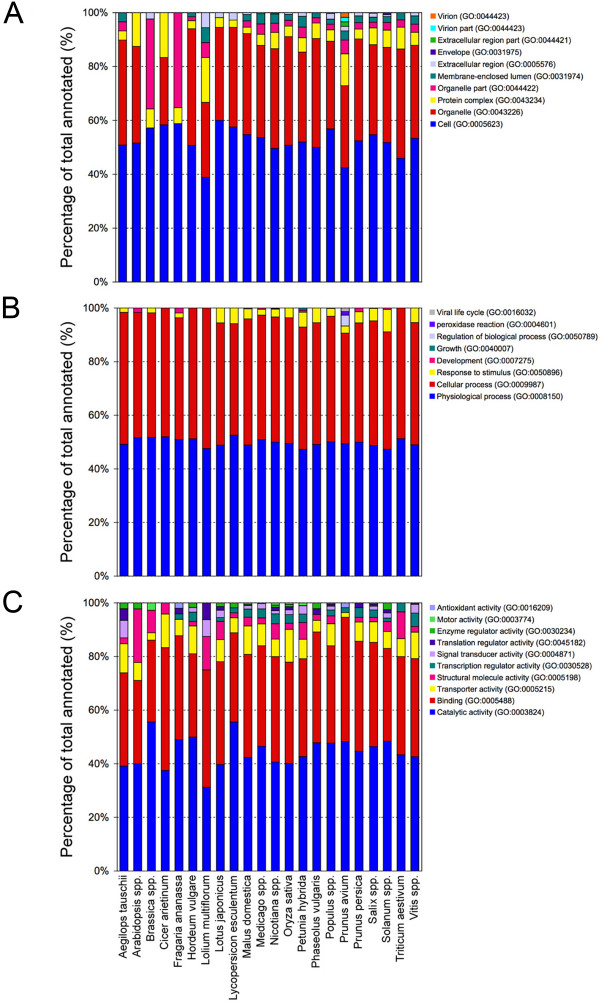
**Level 2 cDNA-AFLPs GO classification by single organism**. GOslim classification of annotated cDNA-AFLP sequences sorted by organisms (genus/species) and analyzed at level 2 for the three main GO vocabularies: cellular component (A), biological process (B) and molecular function (C).

The GO terms obtained by the annotation procedure were mapped to a plant specific GOslim to generate a more concise annotation to be used for comparative analyses as reported in similar researches [44-46]. In the present study the 'goslim_plant.obo' developed by Mundodi S. The FTP directory of the Genome Databases Group, Department of Genetics, Stanford University School of Medicine was used (please see Availability & requirements for more information) [47]. The GOslim classification of annotated cDNA-AFLP sequences is reported in Figures 3 and 4. Level 2 annotation was chosen because representative of all retrieved annotation, and a comparison with the annotations of Arabidopsis and rice ESTs was possible. Among the annotated sequences, 479 were attributable to roots, 903 to stems plus leaves, 125 to flowers, 342 to fruits and 114 to seeds. For the remaining 780 (28% of the total annotated), no information on the plant tissue or organ source was available among the features of the database entries.

Concerning the annotations of sequences grouped by organs and tissues (Fig. 3, left panel), it is worth noting that the proportion of fruit records with catalytic and transporter activities was significantly higher (1.5- and 2-fold, respectively) than that observed for other organs (χ^2 ^≥ 12.969, *P *≤ 0.0002 and χ^2 ^≥ 7.178, *P *≤ 0.0054, respectively), whereas the proportion of sequences related to 'binding' was much lower (about 5-fold) than that calculated for roots, leaves and stems, flowers and seeds (χ^2 ^≥ 94.173, *P *≤ 0.0001). The annotation of sequences according to the botanic family was also performed (Fig. 3, right panel). The relative proportions of sequences associated with the two most abundant categories of CC ('cell' and 'organelle'), BP ('physiological process' and 'cellular process') and MF ('catalytic activity' and 'binding') proved to be very similar each other, with relative differences smaller than 5%. These findings indicate that GO annotation data gained for AFLP-derived cDNA sequences in plants are highly concordant, when compared among botanic families, but also significantly divergent, when referred to different plant organs or tissues.

The classification of annotated cDNA-AFLP sequences at level 2 is summarized in Figure 4 for each of the 22 taxonomic entities/organisms analyzed in this study and sorted by genus or species. The proportion of annotated sequences for each organism varied from 26.4% of *Nicotiana *spp. to 4.7% of *Lolium multiflorum*. Consistent differences were pointed out among species for GO terms belonging to the three subvocabularies.

### GO multilevel analysis of AFLP-derived cDNA sequences

All cDNA-AFLP sequences sorted by organs and families were also analyzed using a multilevel procedure. This further investigation enabled to find out the lowest node per branch of the DAG (Directed Acyclic Graphs, *i.e*. the hierarchical representation of the gene ontology) that fulfils the filter condition, *e*.*g*. will find all the lowest nodes with the given number of sequences or score value (see Additional files 4, 5, 6, 7, 8, 9).

The most represented terms among the 1,485 belonging to CC for plant organs were 'membrane' for fruits (32.9%) and roots (26.3%), and 'plastid' for seeds (30.4%), flowers (29.6%), and leaves and stems (23.7%). Sequences associated with 'cytoplasmic membrane-bound vesicle', 'intracellular organelle part' and 'membrane part' were only found in leaves and stems. The most represented BP terms among the 1,565 for plant organs were 'transport' in seeds (36.1%%), fruits (26.8%) and roots (21.7%), 'secretory pathway' (18.0%) in leaves and stems, 'amino acid and derivative metabolism' (15.9%) in flowers. Finally, the most represented terms among the 1,350 belonging to MF for plant organs were 'hydrolase activity' in roots (32.5%) and seeds (30.8%), 'oxidoreductase activity' (20.1%) in leaves and stems, 'nucleotide binding' in flowers (30.4%) and fruits (28.3%). Sequences associated with 'oxidoreductase activity', 'ATP binding', 'transition metal ion binding', 'protein kinase activity' and 'peptidase activity' were only recorded for leaves and stems. Conversely, sequences related to 'hydrolase activity' and 'nucleotide binding' were found over all organs, except for leaves and stems (see also Additional files 4, 5, 6).

Regarding the by-family functional analysis at multiple levels, again an overall agreement in most represented GO terms was observed. Concerning the CC, 'membrane' was the most abundant term in all families, except for the *Solanaceae *where 'protein complex' was the most frequent. 'Plastid' and 'mitochondrion' were also abundant in the *Fabaceae *and *Poaceae*. For the BP ontology, the most numerically represented categories related to 'transport' over all families, varying between 16.7% of *Brassicaceae *to 29.7% of *Poaceae*. Among the other GO terms, the most abundant within family were 'cell organization and biogenesis' in *Solanaceae*, 'protein modification' in *Fabaceae*, *Rosaceae *and *Solanaceae*, 'biosynthesis' in *Fabaceae*, 'carbohydrate metabolism' in *Rosaceae *and *Salicaceae*, 'amino acid and derivative metabolism' in *Fabaceae*, *Poaceae *and *Salicaceae*. Among the MF terms, 'hydrolase activity' and 'nucleotide binding' included the highest proportion of sequences over all families. In particular, sequences associated with the former spanned from 19.0% of *Solanaceae *to 29.0% of *Fabaceae*, whereas those associated with the latter were around 25% in most families. Moreover, cDNA-AFLP profiling proved to assay expressed sequences related to 'kinase activity' with very similar efficiency (about 11%) in each of the seven families analyzed in this study (for details see Additional file 7, 8, 9).

### Annotation index (*Ai*) estimates of AFLP-derived cDNA sequences

An annotation index (*Ai*) was developed to describe the information content of AFLP-derived cDNA sequence collections on the basis of the GO annotations.

Computational simulations performed with random sets of 1,000 cDNA-AFLP sequences revealed that *Ai *values can range between 0.2 and 3.0 (data not shown), depending on the proportion of sequences associated with combinations of terms belonging to one, two or all the three GO vocabularies. Annotations indexes were estimated for plant organs/tissues and botanic families in order to perform comparisons. Sequences related to seeds and fruits were those displaying, respectively, the lowest and highest *Ai *with almost a two-fold difference, *i*.*e*. 0.70 vs. 1.32 (Table  4). Concerning botanic families, *Ai *ranged from 0.68 of *Solanaceae *to 1.51 of *Vitaceae*, with an average value for the total set of sequences of 0.93.

**Table 4:** Annotation indexes.

Group	*Ai*
Root	1.01
Stem + Leaf	1.05
Flower	0.86
Fruit	1.32
Seed	0.70
	
*Brassicaceae*	0.78
*Fabaceae*	1.14
*Poaceae*	1.22
*Rosaceae*	1.26
*Salicaceae*	1.04
*Solanaceae*	0.68
*Vitaceae*	1.51

Total	0.93

Annotation index (*Ai*) estimates for the groups of cDNA-AFLP-derived sequences analyzed.

### GO comparison between dicots and monocots AFLP-derived cDNA sequences

**Figure 5:**
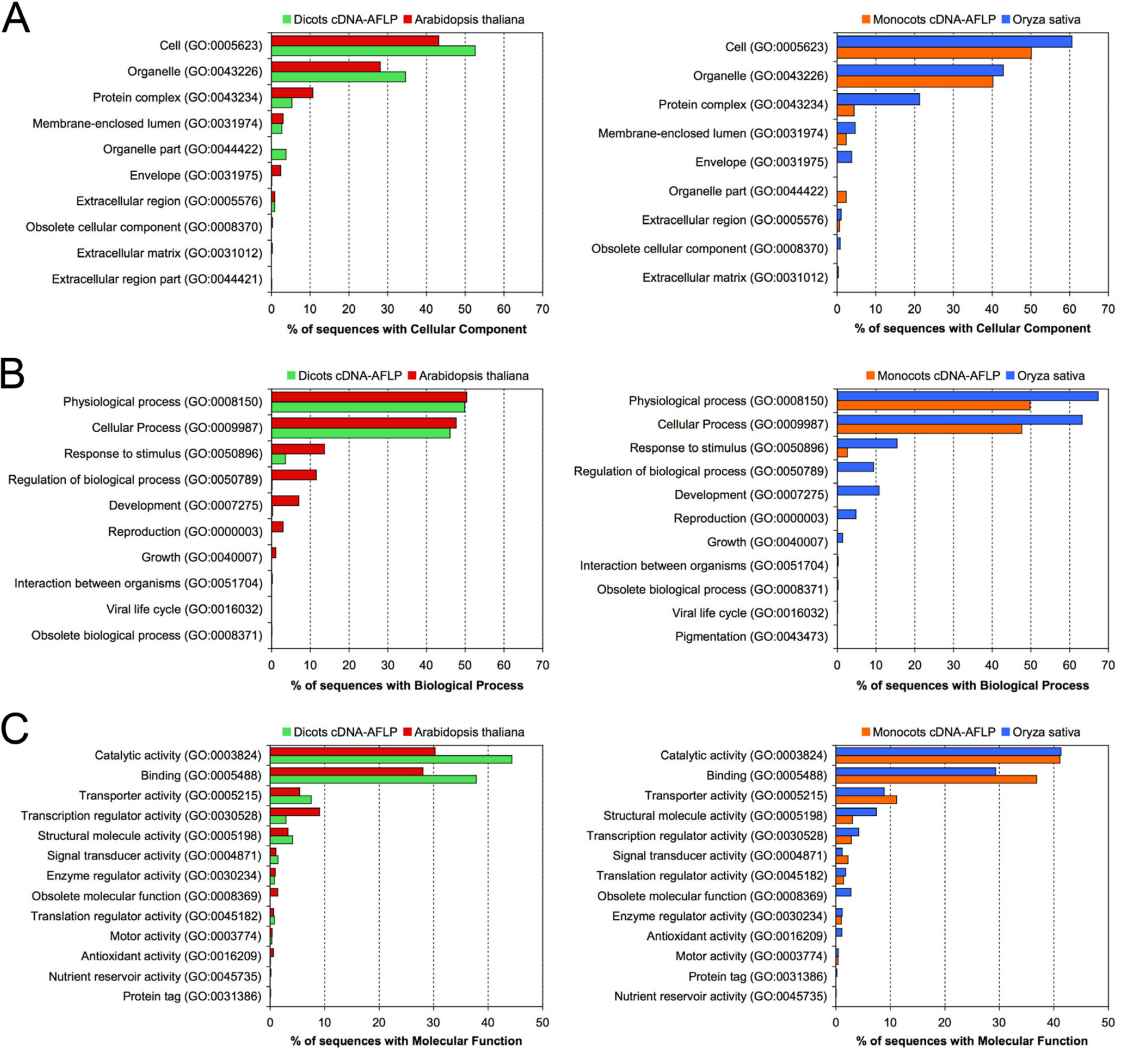
**Level 2 cDNA-AFLPs GO classification in monocots and dicots**. GOslim classification of cDNA-AFLP sequences subgrouped in monocots and dicots, and analyzed at level 2 for the three main GO vocabularies: cellular component (A), biological process (B) and molecular function (C). Output data were then compared to all annotated ESTs of *Arabidopsis thaliana *and *Oryza sativa*, respectively.

In order to study the functional content of cDNA-AFLP sequences in relation to expressed sequences in model organisms, a level 2 GOSlim classification of all the cDNA-AFLP records was performed by splitting the sequence dataset into monocots and dicots and by comparing the two subgroups with all annotated ESTs (please see Availability & requirements for more information) of *Oryza sativa *and *Arabidopsis thaliana*, respectively (Fig. 5).

The comparison between dicot cDNA-AFLPs and Arabidopsis EST sequences revealed substantial differences for the relative frequencies of the most represented GO terms associated with CC and MF ontologies. In particular, the CC categories of 'cell', 'organelle' and 'protein complex' included 43.1%, 28.1% and 10.7% of Arabidopsis EST sequences against 52.6%, 34.6% and 5.3% of dicot cDNA-AFLP sequences, respectively (all pairwise comparisons in terms of absolute frequencies were significant or highly significant, being χ^2 ^≥ 9.148 and *P *≤ 0.0025). Even the GO terms related to 'organelle part' and 'envelope' were strongly differentiated between Arabidopsis and dicot species. The two major GO categories associated with BP, which were 'physiological process' and 'cellular process', showed to be similarly represented in dicot species and Arabidopsis, being their proportions equal to 49.8% vs. 50.4% and 46.1% vs. 47.7%, respectively. A large difference was observed for the term 'response to stimulus', which was assigned to 13.6% of Arabidopsis sequences and only to 3.6% in dicot cDNA-AFLPs (χ^2 ^= 162.02, *P *= 1.3e-45). Four additional GO term categories, 'regulation of biological process', 'development', 'reproduction' and 'growth' were found at 11.5%, 7.1%, 3.0% and 1.1%, respectively, in Arabidopsis and at much lower frequencies in the dicot species (< 0.3%). Regarding MF terms, highly significant differences were found for the two major categories, 'catalytic activity' and 'binding': the former was represented by 30.2% and 44.3% of the sequences, respectively, in Arabidopsis and dicot species, whereas the latter was 28% in Arabidopsis and 37.8% in the dicots (χ^2 ^≥ 55.009, *P *≤ 1.68e-13 and χ^2 ^≥ 12.391, *P *≤ 4.44e-5, respectively). A marked difference was also found for the category 'transcription regulator activity' (9.1% of Arabidopsis vs. 2.9% of dicots, with χ^2 ^= 102.12 and *P *= 1.71e-29).

The comparison between monocot cDNA-AFLPs and rice EST sequences revealed also consistent differences for some of the most frequent GO terms. While in the CC branch the two major categories of 'cell' and 'organelle' were similarly represented, with 50.2% vs. 60.6% and 40.2% vs. 42.9%, respectively (χ^2 ^= 6.158, *P *= 0.012 and χ^2 ^= 1.894, n.s., respectively), a marked difference was observed for 'protein complex', being the frequency of sequences associated with this category equal to 21.3% in rice and 4.4% in the monocot sequences (χ^2 ^= 61.677, *P *= 2.02e-19). Regarding the BP, the three major GO terms proved to be much more frequent in rice ESTs than in monocot cDNA-AFLP sequences, being equal to 67.4% vs. 49.8% for 'physiological process', 63.2% vs. 47.6% for 'cellular process' and 15.5% vs. 2.6% for 'response to stimulus', with highly significant differences (χ^2 ^≥ 156.1, *P *≤ 4.39e-35), 10.8% vs. 0% for 'development', and 9,4% vs. 0% for 'regulation of biological process'. Non-significant differences were observed for the GO terms belonging to the MF vocabulary, except for the category of 'binding' (χ^2 ^= 12.601, *P *= 0.000045) (Fig. 5).

## Discussion

### Experimental procedures adopted for GO analysis of AFLP-derived sequences

**Figure 6:**
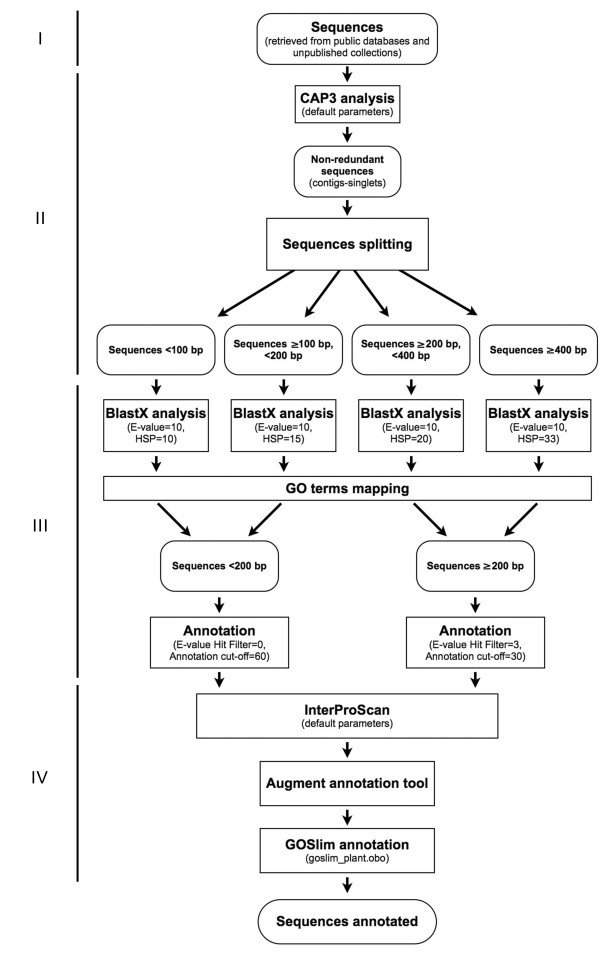
**The experimental pipeline**. Experimental steps and statistical parameters adopted for the bioinformatic analyses of AFLP-derived sequences. Four main steps were followed to achieve the GO classification of AFLP-derived sequences: I. Data recovery from sequence databases; II. Preliminary selection of nucleotide records; III. Basic annotation; IV. Enrichment and refinement of annotation terms.

The experimental steps and statistical parameters adopted for AFLP-derived sequence analysis are critical for obtaining reliable ontology data and deserve a specific mention. Four main steps were followed to achieve a reliable GO classification of AFLP-derived sequences: I. Data recovery from sequence databases; II. Preliminary selection of nucleotide records; III. Basic annotation; IV. Enrichment and refinement of annotation terms (Fig. 6).

The retrieval of AFLP-related sequences from NCBI was performed by searching for the keywords "AFLP and viridiplantae" and "cDNA-AFLP and viridiplantae" and subsequent manual scoring to verify the results. The manual step was required because the keywords indicating the AFLP origin were not always present in the record fields. For our purpose, this seemed to be a noticeable limitation of the GenBank database, probably due both to an unintentional inaccuracy in submitting sequences and an insufficient stringency in accepting a new record without any precise indication of its origin. As a general remark, nucleotide NCBI database curators should consider requesting additional and more precise information about the origin of the records to be submitted. In the case of AFLP-derived sequences, at least the restriction enzymes and selective primer combinations used in the experiments should be added in the 'features' or 'comment' fields, as already done by several submitters. The presence of redundant sequences (20% on average) was a further known limitation, caused mainly by a poor evaluation of the records already present in the public databases at the time of a new submission. However, possible polymorphisms, even at the single nucleotide level (SNPs), have to be always considered as highly informative and carefully evaluated for their reliability.

The basic annotation obtained by retrieving GO terms from BlastX matches was enriched and refined using three main strategies integrated in the Blast2GO software: i) InterProScan, ii) ANNEX, and iii) GOslim simplified annotation. Databases of protein domains and functional sites have become vital resources for the prediction of protein functions. InterProScan [48] combines different protein signature recognition methods allowing searches against independent databases and an easy recovery of the corresponding codes to be automatically coupled to GO terms annotation. This tool allowed an average 8% increase in the overall annotated sequences (data not shown). This value is in the range of observed improvement in blast-based GO annotation by InterPro (Goetz, personal communication) and shows the important contribution of motif-based annotation in GO terms enrichment as well as the basic importance of an integrated approach to the annotation of unknown nucleotide records. A refinement of the GO terms was obtained by means of the ANNEX function (please see Availability & requirements for more information). Using the original GO structure, ANNEX locates parent-offspring relationships between the annotations. Afterwards, ANNEX suggests new BP and CC annotations as well as implicit ones deduced from univocal relationships between GO terms from the different GO categories providing on average 10–15% increase in functional terms. In other words, given a molecular function, this tool identifies biological processes where the molecular functions are involved and the cellular components where they are active. For a detailed description of the ANNEX procedure, see [49].

In the last step of the annotation procedure, a simplified version of the full ontologies (*i*.*e*. the GOslim) was adopted [44-46]. GOslims are cut-down versions of the full ontologies composed by high-level selected terms, or nodes, each one including subsets of the terms of the whole GO. They give a broad overview of the ontology content without the detail of the specific fine-grained terms. GOslims are created by users according to their needs, and may be specific to species or to particular areas of the ontologies. GO provides a generic GOslim which, like the GO itself, is not species-specific, and which should be suitable for most purposes such as the comparisons made in this research. The adoption of the 'goslim_plant.obo' GOslim allowed a visible improvement of the annotation in terms of a reduced fragmentation of the GO categories and a more plant-specific terminology. Indeed, all the GO terms specifically related to CC, MF and BP typical of mammals and/or unicellular organisms were removed or replaced with plant-specific terms.

The Blast2GO software was shown to be very effective in every step of the annotation procedure particularly with respect to the integrated tools that allowed a great time saving during the enrichment and refinement steps. This tool offers a suitable platform for functional genomics research in non-model species and it also allows monitoring and comprehension of the whole annotation and analysis steps. Some minimal limitations were found during the elaboration of results, in particular concerning the generation of graphics. However, future implementations may allow the Blast2GO software to be one of the most powerful tools for the annotation of unknown sequence pools.

### Gene expression analysis by means of cDNA-AFLP technique

The generation of transcriptional profiles has numerous applications in plant biology, including the identification of tissue-specific or developmental stage-specific, reproduction-related and stress-induced transcripts [26,27,29,31,32,37,50-57]. Many new techniques have been designed in recent years for this purpose, such as microarrays [58], allowing a serial genome-wide expression profile of thousands of genes to be performed in a single experiment. Though powerful, this approach is often really expensive and can be readily applied only to model species for which significant information about the coding sequences is available. Moreover, because rarely expressed transcripts are usually missing from cDNA libraries due to over-representation of abundant messengers, microarrays could fail to detect transcripts that are rare but fundamental for certain traits. Library enrichment approaches, such as library subtraction or normalization [59,60], could also be adopted to catch low-copy mRNAs, although time-consuming and cost-effective. On the other hand, the cDNA-AFLP technique has become one of the most robust solutions for differential display and it has been successfully applied in several quantitative gene expression studies [21,24,40,61,62], even in less investigated species for which sequence information is not available. Therefore, after ten years from its first applications in plants, cDNA-AFLP represents an irreplaceable tool for transcriptome profiling analysis that can be utilized for multifactorial genomics of any species. The utility of cDNA-AFLP in functional genomics and systems biology research in plants can be further implemented by serial annotation analyses of species/organ/tissue-specific repertories of transcript-derived fragments, starting from the data provided in the present study. Moreover, the annotations have to be continuously updated, since further information is available by functional characterization of the most interesting sequences. In this way, annotation data may allow to set up a forecasting model according to which the cDNA-AFLP experiments can be tuned to target specific gene classes, and therefore, retrieving gene sequences enriched with the desired functional categories.

### BlastX querying, GO terms mapping and annotation of transcript sequences recovered by cDNA-AFLP analysis

The relatively high number of specific studies carried out in recent years using cDNA-AFLP as a differential display technique does not match a correspondingly high number of sequences retrievable from public databases. The total number of plant AFLP-derived EST entries was shown to be as low as 7,806, successively reduced to 6,748 because of the 23% of redundancy. Moreover, only 22 plant species/genera were represented over all records, with *Solanaceae *being the most numerous family.

A total of 4,332 cDNA-AFLP sequences revealed structural homology and significant similarity with deposited records, allowing to retrieve as many as 11,409 GO terms. The subsequent validation performed with the integrated function of the Blast2GO software enabled the annotation of 2,743 cDNA-AFLP sequences, each with 1 to 26 GO terms. Despite the important role of cereals and related research, the highest number (90%) of AFLP-derived ESTs with Blast matches was found in the *Rosaceae *family, immediately followed by the *Vitaceae *(84%), the latter being mostly represented by grape sequences. These two taxonomic groups have been subjected to intense research activities in the last few years, supported by worldwide initiatives such as the GDR (Genomic Database for *Rosaceae*, [63]) at Clemson University (USA), ESTree consortium in Italy (please see Availability & requirements for more information) which is focused mainly on peach, International Grape Genome Program (please see Availability & requirements for more information) in United States and the Franco-Italian grape genome sequencing project (please see Availability & requirements for more information). The enrichment of database information resulting from such collaborative researches may be in part responsible of the availability of Blast matches for *Rosaceae *and *Vitaceae *sequences. For a parametric description of sequence annotability, a dedicated index (*Ai*, Annotation index) was developed in the present research and calculated for the most interesting sequence subsets. Therefore, the *Ai *computed for *Rosaceae *and *Vitaceae *was as high as 1.26 and 1.51, respectively (Table 4). Taking into account the other botanic families, some interesting results were pointed out concerning the annotability of sequences. In particular, *Solanaceae *family strangely showed the lowest percentage (31%) of annotated sequences, with *Ai *as low as 0.68 (see Fig. 2 and Table 4), despite the high number of entries present in the databases and the fact that species belonging to this taxonomic group, such as tomato, tobacco and petunia, are widely used as models.

Considering the information content of sequences classified according to the organ, tissue or part of the plant from which they were previously isolated, some interesting results were evidenced from the analysis of data distribution (Fig. 2). The highest percentage (90%) of sequences with Blast results was found for cDNA-AFLP entries isolated from fruits, that were also the plant organs with the highest percentage of annotated sequences, with an *Ai *as high as 1.32 (Table 4). This result agrees with those found for the *Rosaceae *family, to which most of the fruits such as peach, apple, pear, plum, apricot, and cherry belong, as well as for the species included in the *Vitaceae *taxonomic group, such as grape. Moreover, the abundance of GO terms associated with carbohydrate, protein and amino acid metabolism, and catalytic as well as transporter activities may reflect the cellular and biochemical events occurring during fruit ripening. Despite the importance of seed development in cereals and legumes, and the amount of studies dedicated to its understanding, sequences related to seeds were those with the lowest percentage of entries with blast hits (51%) and annotation (29%), displaying an *Ai *= 0.70 (Table 4).

On the whole, our findings suggest that the annotability of a set of sequences from a specific plant organ or a particular taxonomic group is not a direct consequence of the amount of research efforts dedicated to studying that organ or group. In fact, the GO annotations of genes are very often transferred from very phylogenetically distant organisms.

The GO characterization and annotation of AFLP-derived sequences allowed us to retrieve basic information on the gene function/s in crop species and their organs/tissues. In particular, examination of thousands of EST clones enabled certain inferences to be made on the potentials and drawbacks of AFLP technology for mRNA profiling and differential display gene cloning. Although the different number of sequences retrieved in databases for plants and organs might have biased some of the descriptive statistics, the overall GO is consistent with the existence of AFLP technology features exploitable across angiosperm. The representativeness of sequence samples and goodness of statistical results over all taxonomic groups are supported by bootstrap analysis. The calculations were performed using the 2,235 singlets of *Nicotiana *spp. with a number of non-overlapping random replicates of 32, 15 and 10 each formed by 70, 149 and 224 sequences, respectively. The bootstrap test was carried out on *Nicotiana *because it was the most numerous genus. The proportions of annotated sequences for each GO vocabulary and over all GO terms per vocabulary proved to be very similar in all replicates with standard deviations lower than 5% in most cases (see Additional File 10). This finding demonstrates the high reliability and reproducibility of gene annotation results for the 22 organisms analyzed in this study. As a consequence, EST repertories equal to or larger than 100 cDNA-AFLP clones can be considered sufficient to obtain a sequence information content representative of the main GO categories.

In this context, it is worth noting that cDNA-AFLP profiling proved to assay expressed sequences related to 'kinase activity' with very similar efficiency in each of the seven families analyzed in this study. It may be deduced that this gene family shows distinctive conserved characteristics so that its members can be repeatedly detected by the cDNA-AFLP technique with a quite constant probability in all plant species.

A GO classification of all cDNA-AFLP records was performed by splitting the sequence dataset into monocots and dicots and by comparing the two subgroups with all annotated ESTs of *Arabidopsis thaliana *and *Oryza sativa*. Although available molecular data may be biased by the experimental methodologies and plant materials, our overall GO results suggest that in a given species and for a given organ or tissue EST repertories developed by construction and screening of cDNA libraries include a sequence pool information content different from and/or complementary to that of TDF (Transcript-Derived Fragment) collections generated by cDNA-AFLP profiling and differential display. Since functional data supplied by AFLP-derived sequences do not fully overlap the functional data obtained by EST projects, the two technologies most likely allow to probe distinct target genes and to capture distinct transcript subsets from a given part of the plant. Alternatively, the functional information obtained by Blast2GO may have been biased in comparison to the available annotation for rice and Arabidopsis.

The comparison between monocot cDNA-AFLPs and rice EST sequences also revealed interesting results. In this case, an explanation for the deviations documented in specific GO branches and categories might be found in the different goals driving basic research projects and applied breeding activities in model and crop plants.

## Conclusion

In the last ten years, the cDNA-AFLP mRNA profiling was largely adopted and considerable repertories of organ-specific and differentially expressed transcripts are now available in public databases for model, crop and tree species. The evaluation, annotation and classification of AFLP-derived sequences would therefore become crucial for both functional genomics and systems biology research in plants. The possibility of using AFLP-derived tags on cDNA fragments produced directly by sequencing-by-synthesis technologies opens up the possibility of not only identifying very large numbers of expressed genes, but also retrieving large-scale SNP collections.

Our study suggests that a reliable GO characterization of AFLP-derived sequences is based on the optimization of experimental steps and statistical parameters adopted for GO analysis. The Blast2GO software was shown to represent a comprehensive bioinformatics solution for functionally characterizing sequences and data mining on the correspondent annotations based on the GO vocabularies. An exhaustive annotation based on gene products similarity Blast searches would offer a suitable platform for functional genomics, particularly useful in non-model plant species.

Therefore, the utility of AFLP technology in structural and functional genomics in plants can be implemented by GO annotation analyses species/organ/tissue-specific repertories of transcriptome-derived fragments. Our suggestion is that AFLP-derived sequences should be systematically subjected to GO annotations before their submission to NCBI databases so that a publicly available information based on yearly larger plant EST collections could be periodically released to the GO Consortium and retrieved by other researchers when searching the GenBank.

## Methods

### cDNA-AFLP sequences

Nucleotide sequences of 7,806 ESTs (Expressed Sequence Tags) isolated by cDNA-AFLP differential mRNA display and AFLP-based mRNA profiling techniques [21,24] were retrieved from both NCBI databases (National Center for Biotechnology Information, please see Availability & requirements for more information) and unpublished EST collections. Sequences were manually filtered and only those with specific annotations proving the actual AFLP derivation were selected for the following analyses (Table 1).

Redundant cDNA-AFLP sequences were clustered and contigs assembled using the CAP3 server of the GDR (Genome Database for Rosaceae) website (please see Availability & requirements for more information; [66]) with the default parameters. A total of 6,679 records were obtained for the following analyses and grouped according to their taxonomy (*i*.*e*. species, genus and family) and the plant tissue from which they had been previously isolated (*i*.*e*. root, leaf and stem, flower, fruit, seed). All the contigs along with singlets were used to search databases using Blast.

### GO annotation using Blast2GO

Blast analyses were performed using Blast2GO software v1.3.3 (please see Availability & requirements for more information; [42,67]). Briefly, Blast2GO uses Blast with a user-defined threshold to find similar sequences from the NCBI NRPD (nr database). Publicly available database cross-reference files are used to look up GO association files and retrieve GO annotations for the Blast matches. Databases and files used in the present research were those publicly available on February, 1^st ^2007. Blast2GO assigns GO annotations to the query sequence by pooling the retrieved GO terms and determining the most specific annotations based on an annotation rule (AR). The AR works by weighting GO evidence codes for each GO term retrieved (defaults weights: IDA = 1.0; IMP = 1.0; IGI = 1.0; IPI = 1.0; IEP = 1.0; TAS = 0.9; NAS = 0.9; IC = 0.9; ISS = 0.9; IGC = 0.9; RCA = 0.9; IEA = 0.7; ND = 0.5; NR = 0.5). The user can select only GO terms greater than a specified AR threshold.

BlastX algorithm was used with different parameters according to the length of the query sequence by defining four ranges, as shown in Figure 6: 0–99 bp, 100–199 bp, 200–399 bp, ≥ 400 bp. Blast expectation value threshold was constantly set to 10, whereas HSP length cutoff was set to 10, 15, 20 and 33, respectively. This approach allowed high stringency alignments for even short sequences. The default Blast remote server (please see Availability & requirements for more information) was used to achieve the most updated database matches.

The Mapping tool of Blast2GO software v1.3.3 was used to obtain GO information from retrieved database matches. Annotation of all sequences was performed with different parameters on two ranges of length, 0–199 bp and ≥ 200 bp. Pre-e-value Hit Filter was set to 0 and 3, respectively, and GO weight constantly to 5.

The similarity threshold was set at 60% and 30% for sequences < 200 bp and ≥ 200 bp, respectively, to allow better matches for shorter sequences. Subsequently, InterProScan [47] was performed to find functional motifs and related GO terms by using the specific tool implemented in the Blast2GO software with the default parameters. Finally, the 'Augment Annotation by ANNEX' function was used to refine annotations (please see Availability & requirements for more information; [48]). The GOslim 'goslim_plant.obo' was used to achieve specific GO terms by means of a plant-specific reduced version of the Gene Ontology (please see Availability & requirements for more information). This approach is summarized in Figure 6.

### Diagrams and graphical representations

The output data of the Blast2GO software were exported in text format, imported into Microsoft Excel spreadsheets, and used to generate pie charts. The hierarchical representation of the gene ontology is structured according to different levels, from the top (level 1) parents corresponding to the three main GO categories (cellular component, biological process, molecular function) to the lowest more specialized child terms (level 2, 3, 4, etc.). In the present research, GO annotated datasets were represented at level 2. This level was chosen because it greatly facilitates comparisons among sequence sets by pointing out the most significant differences. Since Blast2GO allows to perform a multilevel analysis, the GO annotations of cDNA-AFLP-derived sequences were also reported (see Additional files 4, 5, 6, 7, 8, 9) by counting the terms at the lowest node per branch that fulfils the filter parameters (*e.g*. the multilevel tool will find all the lowest nodes with the given number of sequences or score value to be plotted jointly). This different approach may offer a second perspective of the annotation of AFLP records by representing a compromise between specificity and representativeness of sequence records.

The Venn diagram was traced by counting the type of annotation using a Perl script.

### Statistical analyses

Basic statistics were calculated for sequence length (bp), sequence similarity (%) and expectation value (E). In addition to the overall mean values, standard deviation and variation coefficients were computed for the total number of annotated sequences, sorted by either botanic family or plant organism, over the three statistical parameters. A restrictive 68% confidence interval, corresponding to the mean value plus and minus the standard deviation, was also calculated along with lower and upper intervals to describe the first two properties using the most representative sequence datasets, whereas median, minimum and maximum values were used to report the third characteristic.

Differences in terms of observed proportions of annotated and non-annotated AFLP-derived sequences sorted by organs/tissues, botanic families, and organisms (genus/species) were tested using a chi-square contingency test [68] for each GO term across the three GO vocabularies. The statistical significance was then computed by performing a Fisher's exact test [69].

Bootstrapping was used to provide sampling standard deviations and determining confidence intervals for the proportion of annotated sequences over all taxonomic groups. The vast majority of taxonomic groups were represented by at least 70 sequences, with an average number of 229 per species/genus (if the outgroup of *Nicotiana *spp. represented by 2,295 sequences is not taken into account, since it is the most numerous). The calculations were performed using the 2,235 singlets of *Nicotiana *spp. with a number of non-overlapping random replicates of 32, 15 and 10 each formed by a number of sequences of 70, 149 and 224, respectively. This analysis provided a convenient way of making inferences on the reliability and reproducibility of GO results being the number of available sequences highly variable (minimum 34 – maximum 2,295) across the 22 taxonomic groups.

### Annotation index

An annotation index (*Ai*) was developed to describe the information content of transcriptome-derived sequence collections on the basis of the GO vocabularies. This coefficient takes into account the number (N) of annotation terms for CC, BP, and MF, and the number of term combinations of two or all three GO vocabularies. *Ai *can be computed as follows:

$$Ai=\frac{NCC+NBP+NMF+2N(CC+BP)+2N(CC+MF)+2N(BP+MF)+3N(CC+BP+MF)}{TNS}xPAS$$

where TNS corresponds to the total number of sequences and PAS is the proportion of annotated sequences. As a consequence, the ratio between the sum of numbers of simple and combined annotation terms and the total number of sequences gives a "qualitative" information, whereas the proportion of annotated sequences over the total is purely a "quantitative" information. *In silico *simulations performed with sets of 1,000 sequences revealed that *Ai *values can range between 0.2 and 3.0 (data not shown): the higher is the proportion of sequences associated with combinations of terms belonging to two and three GO vocabularies, the higher is the value of annotation coefficient. For instance, when the percentage of sequences associated with only one, a combination of two or all three GO terms is equivalent (*i*.*e*. 33.3%, 33.3% and 33.3%), then the *Ai *value corresponds to 1, 1.5 and 2, respectively with 50%, 75% and 100% of annotated sequences (see Table 4).

## Availability & requirements

FTP directory, Genome Databases Group, Department of Genetics, Stanford University School of Medicine: 

DFCI Plant Gene Indices: 

ANNEX function, The Gene Ontology Annotation Toolbox: 

ESTree consortium: 

International Grape Genome Program: 

Franco-Italian grape genome sequencing project: 

National Center for Biotechnology Information: 

Genome Database for Rosaceae: 

Blast2GO software v1.3.3: 

Default Blast remote server: 

Reduced version of the Gene Ontology: 

## Authors' contributions

AB, GB and GG conceived the study. AB and GG performed the bioinformatic analysis. AC and CB supervised the parts related to the Blast2GO software and the AFLP technology, respectively, giving an essential contribution to the organization of the manuscript. GB wrote the manuscript and AR gave useful comments and suggestions about the experimental procedures as well as the results interpretation. All the authors read and approved the manuscript.

## Supplementary Material

Additional file 1 Number and distribution of plant AFLP records (source: PubMed, years 1994–2007). Click here for file

Additional file 2 Statistics on cDNA-AFLP sequences with BlastX matches sorted by botanic family. Click here for file

Additional file 3 Statistics on cDNA-AFLP sequences with BlastX matches sorted by organism. Click here for file

Additional file 4 Multilevel GO analysis for cellular component ontologies using cDNA-AFLP sequences sorted by plant organs. Click here for file

Additional file 5 Multilevel GO analysis for biological process ontologies using cDNA-AFLP sequences sorted by plant organs. Click here for file

Additional file 6 Multilevel GO analysis for molecular function ontologies using cDNA-AFLP sequences sorted by plant organs. Click here for file

Additional file 7 Multilevel GO analysis for cellular component ontologies using cDNA-AFLP sequences sorted by botanic families. Click here for file

Additional file 8 Multilevel GO analysis for biological process ontologies using cDNA-AFLP sequences sorted by botanic families. Click here for file

Additional file 9 Multilevel GO analysis for molecular function ontologies using cDNA-AFLP sequences sorted by botanic families. Click here for file

Additional file 10 Summary results of the bootstrapping analysis using a variable number of groups and sequences. Click here for file
