# Supplementary figures and images for: Large-scale Gene Ontology analysis of plant transcriptome-derived sequences retrieved by AFLP technology
Source: BMC Genomics. 2008 Jul 24;9:347. doi: 10.1186/1471-2164-9-347 (PMC2515857; doi:10.1186/1471-2164-9-347)

**AFLP records in publications**

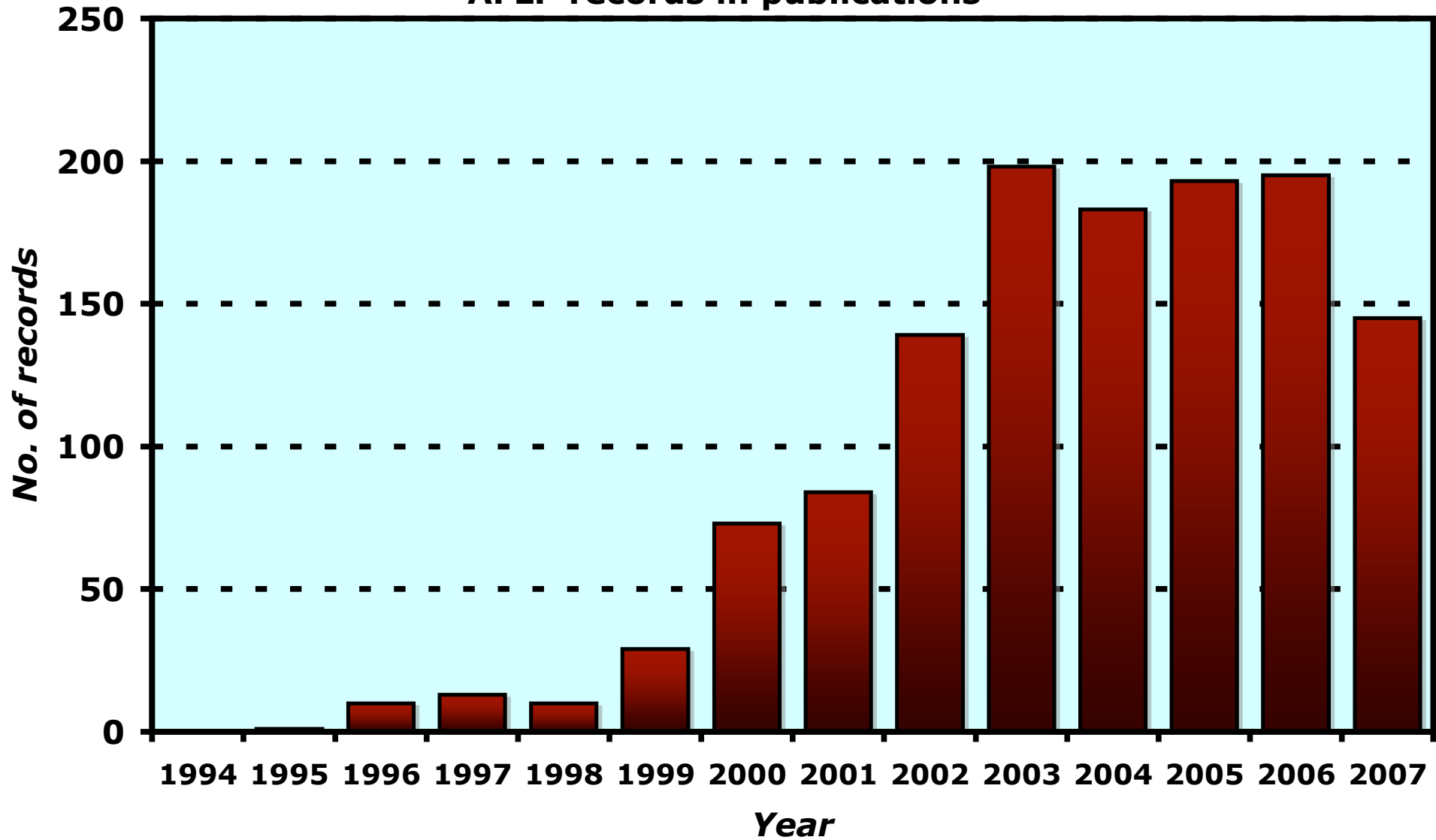

Supplement: Additional file 1 — Number and distribution of plant AFLP records (source: PubMed, years 1994–2007). [file 1471-2164-9-347-S1.pdf]
