# Supplementary material for: Large-scale Gene Ontology analysis of plant transcriptome-derived sequences retrieved by AFLP technology
Source: BMC Genomics. 2008 Jul 24;9:347. doi: 10.1186/1471-2164-9-347 (PMC2515857; doi:10.1186/1471-2164-9-347)
Supplement: Additional file 2 — Statistics on cDNA-AFLP sequences with BlastX matches sorted by botanic family. [file 1471-2164-9-347-S2.doc]

**Additional file 2.** Statistics on cDNA-AFLP sequences with BlastX matches sorted by botanic family.

|  |  | **Length (bp)** | | |  | **Similarity (%)** | |  |  | **E-value** | |
| --- | --- | --- | --- | --- | --- | --- | --- | --- | --- | --- | --- |
| Family | Total No. | No.^1^ | Mean^2^ (conf. int.)^1^ | CV (%)^2^ |  | No.^1^ | Mean^2^ (conf. int.)^1^ | CV (%)^2^ |  | Median^2^ | min^2^ – max^2^ |
| *Brassicaceae* | 200 | 135 | 220 (115 – 326) | 48 |  | 150 | 85 (71 – 98) | 16 |  | 1e-14 | 1e-95 – 1e+00 |
| *Fabaceae* | 634 | 480 | 232 (104 – 359) | 55 |  | 408 | 78 (65 – 92) | 17 |  | 1e-12 | 1e-122 – 1e+00 |
| *Poaceae* | 585 | 512 | 247 (71 – 423) | 71 |  | 411 | 82 (67 – 97) | 18 |  | 1e-15 | 1e-170 – 1e+00 |
| *Rosaceae* | 587 | 407 | 316 (188 – 444) | 40 |  | 396 | 79 (65 – 93) | 18 |  | 1e-25 | 1e-135 – 1e+00 |
| *Salicaceae* | 564 | 410 | 206 (111 – 301) | 46 |  | 379 | 80 (66 – 94) | 17 |  | 1e-11 | 1e-93 – 1e+00 |
| *Solanaceae* | 1,597 | 1,316 | 236 (86 – 385) | 63 |  | 1,025 | 79 (65 – 93) | 18 |  | 1e-11 | 1e-170 – 1e+00 |
| *Vitaceae* | 165 | 116 | 301 (169 – 433) | 44 |  | 99 | 76 (62 – 91) | 19 |  | 1e-19 | 1e-104 – 1e+00 |
| All organisms | 4,332 | 3,376 | 247 (104 – 390) | 58 |  | 2,868 | 80 (65 – 94) | 18 |  | 1e-13 | 1e-170 – 1e+00 |

^1^Refers to the number of sequences included in the 68% confidence interval; ^2^Refers to the total number of sequences.

Abbreviations: conf. int., confidence interval; CV, coefficient of variability; min, minimum; max, maximum.
