# Supplementary material for: Large-scale Gene Ontology analysis of plant transcriptome-derived sequences retrieved by AFLP technology
Source: BMC Genomics. 2008 Jul 24;9:347. doi: 10.1186/1471-2164-9-347 (PMC2515857; doi:10.1186/1471-2164-9-347)
Supplement: Additional file 3 — Statistics on cDNA-AFLP sequences with BlastX matches sorted by organism. [file 1471-2164-9-347-S3.doc]

**Additional file 3.** Statistics on cDNA–AFLP sequences with BlastX matches sorted by organism.

|  |  | **Length (bp)** | | |  | **Similarity (%)** | | |  | **E-value** | |
| --- | --- | --- | --- | --- | --- | --- | --- | --- | --- | --- | --- |
| Organism | Total No. | No.^1^ | Mean^2^ (conf. int.)^1^ | CV (%)^2^ |  | No.^1^ | Mean^2^ (conf. int.)^1^ | CV (%)^2^ |  | Median^2^ | min^2^ – max^2^ |
| *Aegilops tauschii* | 65 | 49 | 228 (160–296) | 30 |  | 39 | 74 (59 – 89) | 20 |  | 1e-09 | 1e-43 – 1e+00 |
| *Arabidopsis* spp. | 116 | 81 | 227 (126 – 328) | 44 |  | 87 | 84 (69 – 98) | 17 |  | 5e-17 | 1e-95 – 1e+00 |
| *Brassica* spp. | 84 | 56 | 211 (99 – 323) | 53 |  | 67 | 86 (72 – 99) | 15 |  | 5e-12 | 1e-93 – 1e+00 |
| *Cicer arietinum* | 35 | 30 | 243 (67 – 419) | 72 |  | 25 | 82 (64 – 99) | 22 |  | 1e-08 | 1e-122 – 1e+00 |
| *Fragaria ananassa* | 60 | 47 | 275 (136 – 415) | 51 |  | 39 | 83 (73 – 93) | 12 |  | 1e-38 | 1e-135 – 1e+00 |
| *Hordeum vulgare* | 60 | 56 | 382 (0 – 800) | 109 |  | 41 | 78 (63 – 94) | 20 |  | 5e-22 | 1e-170 – 1e+00 |
| *Lolium multiflorum* | 25 | 16 | 204 (128 – 280) | 37 |  | 17 | 78 (61 – 95) | 22 |  | 1e-07 | 1e-51 – 1e+00 |
| *Lotus japonicus* | 77 | 71 | 179 (77 – 280) | 57 |  | 53 | 83 (70 – 95) | 15 |  | 1e-07 | 1e-86 – 1e+00 |
| *Lycopersicon esculentum* | 125 | 92 | 171 (95 – 247) | 44 |  | 81 | 83 (72 – 95) | 14 |  | 1e-12 | 1e-78 – 1e+00 |
| *Malus domestica* | 407 | 276 | 328 (197 – 460) | 40 |  | 278 | 78 (64 – 93) | 18 |  | 1e-24 | 1e-109 – 1e+00 |
| *Medicago* spp. | 454 | 323 | 247 (116 – 378) | 53 |  | 297 | 80 (67 – 94) | 17 |  | 1e-13 | 1e-116 – 1e+00 |
| *Nicotiana* spp*.* | 1,200 | 1,029 | 236 (78 – 394) | 67 |  | 787 | 78 (63 – 93) | 19 |  | 1e-10 | 1e-170 – 1e+00 |
| *Oryza sativa* | 386 | 251 | 230 (111 – 349) | 51 |  | 278 | 85 (72 – 99) | 16 |  | 1e-15 | 1e-98 – 1e+00 |
| *Petunia hybrida* | 115 | 80 | 275 (162 – 386) | 41 |  | 75 | 80 (65 – 93) | 17 |  | 1e-16 | 1e-75 – 1e+00 |
| *Phaseolus vulgaris* | 68 | 45 | 181 (127 – 236) | 30 |  | 44 | 80 (66 – 94) | 17 |  | 1e-08 | 1e-40 – 1e+00 |
| *Populus* spp. | 524 | 380 | 205 (111 – 300) | 46 |  | 345 | 81 (67 – 94) | 17 |  | 1e-12 | 1e-93 – 1e+00 |
| *Prunus avium* | 56 | 37 | 256 (158 – 354) | 38 |  | 40 | 80 (69 – 92) | 15 |  | 5e-19 | 1e-50 – 1e+00 |
| *Prunus persica* | 64 | 46 | 329 (237 – 422) | 28 |  | 41 | 77 (61 – 92) | 20 |  | 5e-32 | 1e-78 – 1e+00 |
| *Salix* spp*.* | 40 | 27 | 217 (122 – 310) | 43 |  | 25 | 76 (61 – 91) | 20 |  | 5e-06 | 1e-62 – 1e+00 |
| *Solanum* spp. | 157 | 112 | 255 (125 – 383) | 51 |  | 106 | 80 (67 – 92) | 16 |  | 1e-13 | 1e-109 – 1e+00 |
| *Triticum aestivum* | 49 | 31 | 262 (144 – 380) | 45 |  | 28 | 77 (60 – 93) | 22 |  | 1e-17 | 1e-109 – 1e+00 |
| *Vitis vinifera* | 165 | 116 | 301 (169 – 433) | 44 |  | 99 | 76 (62 – 91) | 19 |  | 1e-19 | 1e-104 – 1e+00 |

^1^Refers to the number of sequences included in the 68% confidence interval; ^2^Refers to the total number of sequences.

Abbreviations: conf. int., confidence interval; CV, coefficient of variability; min, minimum; max, maximum.
