# Supplementary material for: Large-scale Gene Ontology analysis of plant transcriptome-derived sequences retrieved by AFLP technology
Source: BMC Genomics. 2008 Jul 24;9:347. doi: 10.1186/1471-2164-9-347 (PMC2515857; doi:10.1186/1471-2164-9-347)
Supplement: Additional file 4 — Multilevel GO analysis for cellular component ontologies using cDNA-AFLP sequences sorted by plant organs. [file 1471-2164-9-347-S4.doc]

**Additional file 4**. Multilevel GO analysis for cellular compartment ontologies using cDNA-AFLP sequences sorted by plant organs.

| Cellular compartment ontology | | Plant organs | | | | |
| --- | --- | --- | --- | --- | --- | --- |
| GO Terms | GO Codes | Root | Leaf + Stem | Flower | Fruit | Seed |
| Plastid | 0009536 | 75 | 176 | 21 | 57 | 28 |
| Mitochondrion | [0005739](http://amigo.geneontology.org/cgi-bin/amigo/go.cgi?view=details&search_constraint=terms&depth=0&query=GO:0005739&session_id=380b1173204688) | 85 | 138 | 16 | 60 | 24 |
| Protein complex | [0043234](http://amigo.geneontology.org/cgi-bin/amigo/go.cgi?view=details&search_constraint=terms&depth=0&query=GO:0043234&session_id=9041b1173204703) | 28 | 130 | 0 | 18 | 6 |
| Cytoplasmic membrane-bound vesicle | 0016023 | 0 | 103 | 0 | 0 | 0 |
| Intracellular organelle part | 0044446 | 0 | 87 | 0 | 0 | 0 |
| Nucleus | [0005634](http://amigo.geneontology.org/cgi-bin/amigo/go.cgi?view=details&search_constraint=terms&depth=0&query=GO:0005634&session_id=7388b1173204846) | 36 | 58 | 9 | 26 | 11 |
| Membrane part | [0044425](http://amigo.geneontology.org/cgi-bin/amigo/go.cgi?view=details&search_constraint=terms&depth=0&query=GO:0044425&session_id=1622b1173204861) | 0 | 52 | 0 | 0 | 0 |
| Membrane | [0016020](http://amigo.geneontology.org/cgi-bin/amigo/go.cgi?view=details&search_constraint=terms&depth=0&query=GO:0016020&session_id=5703b1173204876) | 89 | 0 | 15 | 79 | 23 |
| Intracellular non-membrane-bound organelle | [0043232](http://amigo.geneontology.org/cgi-bin/amigo/go.cgi?view=details&search_constraint=terms&depth=0&query=GO:0043232&session_id=2975b1173204889) | 25 | 0 | 10 | 0 | 0 |
