# Supplementary material for: Large-scale Gene Ontology analysis of plant transcriptome-derived sequences retrieved by AFLP technology
Source: BMC Genomics. 2008 Jul 24;9:347. doi: 10.1186/1471-2164-9-347 (PMC2515857; doi:10.1186/1471-2164-9-347)
Supplement: Additional file 5 — Multilevel GO analysis for biological process ontologies using cDNA-AFLP sequences sorted by plant organs. [file 1471-2164-9-347-S5.doc]

**Additional file 5**. Multilevel GO analysis for biological process ontologies using cDNA-AFLP sequences sorted by plant organs.

| Biological process ontology | | Plant organs | | | | |
| --- | --- | --- | --- | --- | --- | --- |
| GO Terms | GO Codes | Root | Leaf + Stem | Flower | Fruit | Seed |
| Secretory pathway | [0045045](http://amigo.geneontology.org/cgi-bin/amigo/go.cgi?view=details&search_constraint=terms&depth=0&query=GO:0045045&session_id=1247b1173205009) | 0 | 104 | 0 | 0 | 0 |
| Macromolecule biosynthesis | [0009059](http://amigo.geneontology.org/cgi-bin/amigo/go.cgi?view=details&search_constraint=terms&depth=0&query=GO:0009059&session_id=1458b1173205025) | 0 | 78 | 0 | 0 | 0 |
| Electron transport | [0006118](http://amigo.geneontology.org/cgi-bin/amigo/go.cgi?view=details&search_constraint=terms&depth=0&query=GO:0006118&session_id=789b1173205050) | 43 | 72 | 12 | 27 | 0 |
| Cellular carbohydrate metabolism | [0044262](http://amigo.geneontology.org/cgi-bin/amigo/go.cgi?view=details&search_constraint=terms&depth=0&query=GO:0044262&session_id=9750b1173205065) | 0 | 68 | 0 | 0 | 0 |
| Amino acid metabolism | [0006520](http://amigo.geneontology.org/cgi-bin/amigo/go.cgi?view=details&search_constraint=terms&depth=0&query=GO:0006520&session_id=2169b1173205081) | 0 | 67 | 0 | 0 | 0 |
| Protein amino acid phosphorylation | 0006468 | 0 | 49 | 0 | 0 | 0 |
| Proteolysis | [0006508](http://amigo.geneontology.org/cgi-bin/amigo/go.cgi?view=details&search_constraint=terms&depth=0&query=GO:0006508&session_id=431b1173205117) | 0 | 47 | 0 | 0 | 0 |
| Regulation of transcription | 0045449 | 0 | 47 | 0 | 0 | 0 |
| Cell organization and biogenesis | 0016043 | 41 | 46 | 11 | 19 | 6 |
| Protein modification | 0006464 | 51 | 0 | 16 | 40 | 11 |
| Transport | 0006810 | 109 | 0 | 17 | 78 | 22 |
| Response to stimulus | [0050896](http://amigo.geneontology.org/cgi-bin/amigo/go.cgi?view=details&search_constraint=terms&depth=0&query=GO:0050896&session_id=5206b1173205234) | 30 | 0 | 10 | 0 | 0 |
| Biosynthesis | 0009058 | 70 | 0 | 0 | 0 | 0 |
| Transcription | 0006350 | 26 | 0 | 7 | 23 | 0 |
| Carbohydrate metabolism | [0005975](http://amigo.geneontology.org/cgi-bin/amigo/go.cgi?view=details&search_constraint=terms&depth=0&query=GO:0005975&session_id=7801b1173205285) | 49 | 0 | 12 | 33 | 0 |
| Catabolism | [0009056](http://amigo.geneontology.org/cgi-bin/amigo/go.cgi?view=details&search_constraint=terms&depth=0&query=GO:0009056&session_id=4562b1173205305) | 35 | 0 | 10 | 19 | 0 |
| Amino acid and derivative metabolism | 0006519 | 49 | 0 | 21 | 30 | 7 |
| Signal transduction | [0007165](http://amigo.geneontology.org/cgi-bin/amigo/go.cgi?view=details&search_constraint=terms&depth=0&query=GO:0007165&session_id=9056b1173205337) | 0 | 0 | 8 | 0 | 0 |
| Protein biosynthesis | [0006412](http://amigo.geneontology.org/cgi-bin/amigo/go.cgi?view=details&search_constraint=terms&depth=0&query=GO:0006412&session_id=5104b1173205352) | 0 | 0 | 8 | 22 | 7 |
| DNA metabolism | 0006259 | 0 | 0 | 0 | 0 | 8 |
