# Supplementary material for: Large-scale Gene Ontology analysis of plant transcriptome-derived sequences retrieved by AFLP technology
Source: BMC Genomics. 2008 Jul 24;9:347. doi: 10.1186/1471-2164-9-347 (PMC2515857; doi:10.1186/1471-2164-9-347)
Supplement: Additional file 6 — Multilevel GO analysis for molecular function ontologies using cDNA-AFLP sequences sorted by plant organs. [file 1471-2164-9-347-S6.doc]

**Additional file 6**. Multilevel GO analysis for molecular function ontologies using cDNA-AFLP sequences sorted by plant organs.

| Molecular function ontology | | Plant organs | | | | |
| --- | --- | --- | --- | --- | --- | --- |
| GO Terms | GO Codes | Root | Leaf + Stem | Flower | Fruit | Seed |
| Oxidoreductase activity | [0016491](http://amigo.geneontology.org/cgi-bin/amigo/go.cgi?view=details&search_constraint=terms&depth=0&query=GO:0016491&session_id=209b1173205413) | 0 | 131 | 0 | 0 | 0 |
| ATP binding | [0005524](http://amigo.geneontology.org/cgi-bin/amigo/go.cgi?view=details&search_constraint=terms&depth=0&query=GO:0005524&session_id=198b1173205430) | 0 | 113 | 0 | 0 | 0 |
| Transition metal ion binding | 0046914 | 0 | 88 | 0 | 0 | 0 |
| Transporter activity | [0005215](http://amigo.geneontology.org/cgi-bin/amigo/go.cgi?view=details&search_constraint=terms&depth=0&query=GO:0005215&session_id=2857b1173205458) | 51 | 73 | 10 | 42 | 11 |
| DNA binding | 0003677 | 42 | 70 | 8 | 25 | 8 |
| Protein binding | [0005515](http://amigo.geneontology.org/cgi-bin/amigo/go.cgi?view=details&search_constraint=terms&depth=0&query=GO:0005515&session_id=919b1173205487) | 0 | 61 | 9 | 19 | 0 |
| Protein kinase activity | 0004672 | 0 | 49 | 0 | 0 | 0 |
| Peptidase activity | 0008233 | 0 | 48 | 0 | 0 | 0 |
| Hydrolase activity | [0016787](http://amigo.geneontology.org/cgi-bin/amigo/go.cgi?view=details&search_constraint=terms&depth=0&query=GO:0016787&session_id=353b1173205559) | 105 | 0 | 13 | 59 | 20 |
| Nucleotide binding | [0000166](http://amigo.geneontology.org/cgi-bin/amigo/go.cgi?view=details&search_constraint=terms&depth=0&query=GO:0000166&session_id=1437b1173205574) | 89 | 0 | 28 | 67 | 19 |
| Kinase activity | [0016301](http://amigo.geneontology.org/cgi-bin/amigo/go.cgi?view=details&search_constraint=terms&depth=0&query=GO:0016301&session_id=1574b1173205591) | 36 | 0 | 16 | 25 | 7 |
| Structural molecule activity | 0005198 | 0 | 0 | 8 | 0 | 0 |
