# Supplementary material for: Large-scale Gene Ontology analysis of plant transcriptome-derived sequences retrieved by AFLP technology
Source: BMC Genomics. 2008 Jul 24;9:347. doi: 10.1186/1471-2164-9-347 (PMC2515857; doi:10.1186/1471-2164-9-347)
Supplement: Additional file 7 — Multilevel GO analysis for cellular component ontologies using cDNA-AFLP sequences sorted by botanic families. [file 1471-2164-9-347-S7.doc]

**Additional file 7**. Multilevel GO analysis for cellular compartment ontologies using cDNA-AFLP sequences sorted by botanic families.

| Cellular compartment ontology | | Botanic families | | | | | | |
| --- | --- | --- | --- | --- | --- | --- | --- | --- |
| GO Terms | GO Codes | Brassicaceae | Fabaceae | Poaceae | Rosaceae | Salicaceae | Solanaceae | Vitaceae |
| Plastid | 0009536 | 15 | 109 | 132 | 65 | 49 | 139 | 23 |
| Mitochondrion | [0005739](http://amigo.geneontology.org/cgi-bin/amigo/go.cgi?view=details&search_constraint=terms&depth=0&query=GO:0005739&session_id=380b1173204688) | 15 | 88 | 122 | 55 | 36 | 132 | 35 |
| Protein complex | [0043234](http://amigo.geneontology.org/cgi-bin/amigo/go.cgi?view=details&search_constraint=terms&depth=0&query=GO:0043234&session_id=9041b1173204703) | 0 | 25 | 0 | 0 | 0 | 178 | 8 |
| Intracellular organelle part | 0044446 | 0 | 0 | 0 | 0 | 0 | 105 | 0 |
| Nucleus | [0005634](http://amigo.geneontology.org/cgi-bin/amigo/go.cgi?view=details&search_constraint=terms&depth=0&query=GO:0005634&session_id=7388b1173204846) | 0 | 43 | 42 | 29 | 20 | 106 | 8 |
| Membrane | [0016020](http://amigo.geneontology.org/cgi-bin/amigo/go.cgi?view=details&search_constraint=terms&depth=0&query=GO:0016020&session_id=5703b1173204876) | 14 | 88 | 87 | 78 | 70 | 143 | 29 |
| Intracellular non-membrane-bound organelle | [0043232](http://amigo.geneontology.org/cgi-bin/amigo/go.cgi?view=details&search_constraint=terms&depth=0&query=GO:0043232&session_id=2975b1173204889) | 0 | 24 | 0 | 0 | 17 | 110 | 0 |
| Ribosome | 0005840 | 14 | 0 | 25 | 0 | 0 | 0 | 0 |
