# Supplementary material for: Large-scale Gene Ontology analysis of plant transcriptome-derived sequences retrieved by AFLP technology
Source: BMC Genomics. 2008 Jul 24;9:347. doi: 10.1186/1471-2164-9-347 (PMC2515857; doi:10.1186/1471-2164-9-347)
Supplement: Additional file 8 — Multilevel GO analysis for biological process ontologies using cDNA-AFLP sequences sorted by botanic families. [file 1471-2164-9-347-S8.doc]

**Additional file 8**. Multilevel GO analysis for biological process ontologies using cDNA-AFLP sequences sorted by botanic families.

| Biological process ontology | | Botanic families | | | | | | |
| --- | --- | --- | --- | --- | --- | --- | --- | --- |
| GO Terms | GO Codes | Brassicaceae | Fabaceae | Poaceae | Rosaceae | Salicaceae | Solanaceae | Vitaceae |
| Electron transport | [0006118](http://amigo.geneontology.org/cgi-bin/amigo/go.cgi?view=details&search_constraint=terms&depth=0&query=GO:0006118&session_id=789b1173205050) | 0 | 42 | 25 | 31 | 37 | 69 | 0 |
| Cell organization and biogenesis | 0016043 | 8 | 34 | 30 | 19 | 28 | 87 | 7 |
| Protein modification | 0006464 | 6 | 72 | 41 | 47 | 29 | 91 | 16 |
| Transport | 0006810 | 16 | 107 | 114 | 79 | 78 | 179 | 34 |
| Response to stimulus | [0050896](http://amigo.geneontology.org/cgi-bin/amigo/go.cgi?view=details&search_constraint=terms&depth=0&query=GO:0050896&session_id=5206b1173205234) | 0 | 24 | 0 | 20 | 0 | 62 | 0 |
| Biosynthesis | 0009058 | 0 | 66 | 0 | 0 | 0 | 0 | 0 |
| Transcription | 0006350 | 0 | 27 | 29 | 25 | 0 | 62 | 10 |
| Carbohydrate metabolism | [0005975](http://amigo.geneontology.org/cgi-bin/amigo/go.cgi?view=details&search_constraint=terms&depth=0&query=GO:0005975&session_id=7801b1173205285) | 10 | 41 | 29 | 45 | 47 | 69 | 12 |
| Catabolism | [0009056](http://amigo.geneontology.org/cgi-bin/amigo/go.cgi?view=details&search_constraint=terms&depth=0&query=GO:0009056&session_id=4562b1173205305) | 9 | 36 | 0 | 21 | 24 | 0 | 7 |
| Amino acid and derivative metabolism | 0006519 | 10 | 54 | 51 | 40 | 50 | 65 | 14 |
| Protein biosynthesis | [0006412](http://amigo.geneontology.org/cgi-bin/amigo/go.cgi?view=details&search_constraint=terms&depth=0&query=GO:0006412&session_id=5752b1173206958) | 16 | 0 | 35 | 19 | 20 | 69 | 7 |
| DNA metabolism | 0006259 | 0 | 0 | 30 | 0 | 0 | 49 | 0 |
| Nucleic acid metabolism | [0006139](http://amigo.geneontology.org/cgi-bin/amigo/go.cgi?view=details&search_constraint=terms&depth=0&query=GO:0006139&session_id=4975b1173206867) | 5 | 0 | 0 | 0 | 40 | 0 | 0 |
| Generation of precursor metabolites and energy | [0006091](http://amigo.geneontology.org/cgi-bin/amigo/go.cgi?view=details&search_constraint=terms&depth=0&query=GO:0006091&session_id=2458b1173206830) | 9 | 0 | 0 | 0 | 0 | 0 | 10 |
| Lipid metabolism | [0006629](http://amigo.geneontology.org/cgi-bin/amigo/go.cgi?view=details&search_constraint=terms&depth=0&query=GO:0006629&session_id=9573b1173206814) | 7 | 0 | 0 | 0 | 0 | 0 | 0 |
