# Supplementary material for: Large-scale Gene Ontology analysis of plant transcriptome-derived sequences retrieved by AFLP technology
Source: BMC Genomics. 2008 Jul 24;9:347. doi: 10.1186/1471-2164-9-347 (PMC2515857; doi:10.1186/1471-2164-9-347)
Supplement: Additional file 9 — Multilevel GO analysis for molecular function ontologies using cDNA-AFLP sequences sorted by botanic families. [file 1471-2164-9-347-S9.doc]

**Additional file 9**. Multilevel GO analysis for molecular function ontologies using cDNA-AFLP sequences sorted by botanic families.

| Molecular function ontology | | Botanic families | | | | | | |
| --- | --- | --- | --- | --- | --- | --- | --- | --- |
| GO Terms | GO Codes | Brassicaceae | Fabaceae | Poaceae | Rosaceae | Salicaceae | Solanaceae | Vitaceae |
| Transporter activity | [0005215](http://amigo.geneontology.org/cgi-bin/amigo/go.cgi?view=details&search_constraint=terms&depth=0&query=GO:0005215&session_id=2857b1173205458) | 0 | 45 | 57 | 41 | 37 | 70 | 18 |
| DNA binding | 0003677 | 0 | 44 | 48 | 29 | 0 | 101 | 11 |
| Protein binding | [0005515](http://amigo.geneontology.org/cgi-bin/amigo/go.cgi?view=details&search_constraint=terms&depth=0&query=GO:0005515&session_id=919b1173205487) | 0 | 30 | 28 | 28 | 24 | 52 | 8 |
| Hydrolase activity | [0016787](http://amigo.geneontology.org/cgi-bin/amigo/go.cgi?view=details&search_constraint=terms&depth=0&query=GO:0016787&session_id=353b1173205559) | 7 | 105 | 73 | 71 | 52 | 135 | 35 |
| Nucleotide binding | [0000166](http://amigo.geneontology.org/cgi-bin/amigo/go.cgi?view=details&search_constraint=terms&depth=0&query=GO:0000166&session_id=1437b1173205574) | 12 | 96 | 79 | 73 | 53 | 204 | 31 |
| Kinase activity | [0016301](http://amigo.geneontology.org/cgi-bin/amigo/go.cgi?view=details&search_constraint=terms&depth=0&query=GO:0016301&session_id=1574b1173205591) | 5 | 42 | 40 | 32 | 20 | 87 | 13 |
| Structural molecule activity | 0005198 | 15 | 0 | 21 | 0 | 0 | 62 | 0 |
| Nucleic acid binding | [0003676](http://amigo.geneontology.org/cgi-bin/amigo/go.cgi?view=details&search_constraint=terms&depth=0&query=GO:0003676&session_id=7404b1173206790) | 11 | 0 | 0 | 0 | 27 | 0 | 0 |
